# Temporal characterization of hyaluronidases after peripheral nerve injury

**DOI:** 10.1371/journal.pone.0289956

**Published:** 2023-08-24

**Authors:** Mary M. Kasper, Bret Ellenbogen, Yuan Li, Christine E. Schmidt

**Affiliations:** 1 J. Crayton Pruitt Department of Biomedical Engineering, University of Florida, Gainesville, FL, United States of America; 2 Department of Chemistry, University of Florida, Gainesville, FL, United States of America; Universidade de Trás-os-Montes e Alto Douro: Universidade de Tras-os-Montes e Alto Douro, PORTUGAL

## Abstract

Hyaluronic acid (HA) is ubiquitously found in biological tissues and mediates wound healing mechanisms after injury by promoting cell migration and proliferation. With the development of tissue-engineered neural therapeutics, including off-the-shelf grafts for peripheral nerve repair, HA is an attractive material for clinical use because of its various biological roles. HA-based biomaterials have been carefully engineered to elicit specific *in vivo* host responses, however an important design feature that should be considered in these scaffolds is endogenous degradation. Hyaluronidases (HYALs) are the complementary enzymes that are responsible for HA turnover. Although HYAL expression has been widely characterized in various tissues, including the central nervous system, and for different pathologies, there remains a lack of knowledge of HYAL mediated turnover in peripheral nerve tissue. In this work, gene expression of two hyaluronidases, HYAL1 and HYAL2, and HA-binding receptor, CD44, were studied in two injury models: rat sciatic nerve crush and critical gap transection. HYAL2 and CD44 were shown to be upregulated 3 days after crush injury, whereas HYAL1 was upregulated at 3 weeks, which collectively demonstrate temporal patterning of HA breakdown. Additionally, differences were observed between HYAL and HA expression at 3 weeks when compared for both nerve injury models. The activity of HYAL in peripheral nerve tissue was determined to be approximately 0.11 μmol/min, which could be used to further model HA-based biomaterial breakdown for peripheral nerve applications. Overall, this work provides a landscape of HA turnover in peripheral nerve that can be used for future neural applications.

## 1. Introduction

Hyaluronan (HA) plays an important role in wound healing, and consequentially, so do its corresponding hyaluronidases [1]. HA is ubiquitously expressed on the surface of various cell types through CD44 binding and also within the extracellular matrix space where HA mediates many critical processes including cell motility, adhesion, and proliferation [2]. For these reasons, HA-based therapeutics have been widely studied in tissue engineering and regenerative medicine to stimulate wound healing responses [3–6]. In tissue engineered applications, HA-based biomaterials have been used for various applications to stimulate cell motility and for structural scaffolding support, however an important facet to consider with any bulk biomaterial is host integration, mediated by endogenous degradation. Hyaluronidases (HYAL) are complementary enzymes to HA, and mediate HA matrix turnover in healthy tissue [7]. There are six types of hyaluronidases that have been characterized in the human genome (i.e., HYAL1, HYAL2, HYAL3, HYAL4, PH20/SPAM1 and HYALP1), with HYAL1 and HYAL2 being the major types present in a diversity of cell populations within mammalian somatic tissues [8].

The proposed mechanism for HA breakdown by hyaluronidases in somatic tissues includes the initial breakdown of large extracellular HA by HYAL2. HA fragments (~20 kDa) generated at the cell surface are endocytosed into vesicles and transported intracellularly where they are further digested by HYAL1 [8, 9]. For a highly voluminous molecule, HA turnover is extremely rapid, with approximately 30% of all HA being broken down and renewed every day [10, 11]. Thus, a balance between HA degradation and synthesis is critical for tissue homeostasis, especially following injury.

The roles of hyaluronidases and their dysregulation have been implicated in various disease and degenerative states [12]. For example, elevated levels of hyaluronidase have been noted in the serum and tissue of patients with end-stage renal [13] and hepatic diseases [14, 15]. Some disease states reveal contradictions in hyaluronidase trends. For example, hyaluronidases are generally measured as a marker for many types of cancer including bladder [16] and breast [17] cancers, whereas downregulation of hyaluronidases has also been observed in later stage ovarian [18, 19] and endometrial [20] cancers. Thus, understanding the balance between hyaluronidases and HA deposition can be important to developing detection methodologies and therapies for specific tissue pathologies that are less understood.

Overproduction and accumulation of HA degradation products following injury are potentially inhibitory to regeneration in the central nervous system [21]. Hyaluronidases in the central nervous system are upregulated after injury to compensate for increases in HA matrix deposition [21, 22]. Studies in hemorrhage [22] and stroke [21] suggest that HA upregulation after injury is inhibitory to regeneration by activating CD44 and toll-like receptors involved in ischemic events and demyelination [23]. Furthermore, studies suggest that endogenous production or exogenous administration of hyaluronidase promotes better functional recovery following spinal cord injury [24] and hemorrhage [22].

Unlike in the central nervous system, hyaluronidases have yet to be characterized in the peripheral nervous system. Studying the temporal patterning of hyaluronidases may be useful to further elucidating the role of ECM in nerve repair. Additionally, acquiring quantitative information for hyaluronidases and HA in peripheral nerve is useful for specifically engineering HA-based peripheral nerve therapies [25, 26]. Therefore, the work presented in this manuscript is focused on the characterization of HA and hyaluronidases (HYAL1 and 2) after peripheral nerve crush and transection injury. In this work, it was found that hyaluronidase species act in a spatiotemporal manner dictated by their roles in HA catabolism. HA and hyaluronidase expression did not significantly change after nerve crush injury out to 6 weeks, however the data presented suggest that there may be differences in HA turnover between transection and crush injury models. The work presented here not only illustrates changes in hyaluronidase expression after nerve injury, but also provides quantitative enzymatic data that will be used to inform the development of HA-based regenerative scaffolds in future work.

## 2. Materials and methods

### 2.1 Surgical procedures

All surgical procedures were performed with prior approval and in accordance with the University of Florida Institutional Animal Care and Use Committee (IACUC) guidelines (protocol #202011139). Animals were received and acclimated for one week before surgical procedures. Animals were fed a 2918 standard diet and housed in a reverse light cycle room (12 h/12 h) at 20-26°C with two animals per cage.

Lewis rats (male, 8 weeks old, 200–250 g [Charles River]) were randomly assigned to experimental timepoints (crush: 1 day, 3 days, 1 week, 3 weeks, 6 weeks; transection: 3 weeks) and tissue processing treatments using a random number generator. In total, 14 animals were assigned per timepoint with 6 animals assigned to gene expression analysis, 6 animals assigned to HA and HYAL biochemical analysis, and 2 animals assigned to immunohistochemical staining. “Fresh” control animals were naïve, i.e., were not operated on before isolating their tissue samples. All quantitative analyses were blinded.

For the 3-week transection group, a hollow conduit was fabricated as a model for a critical gap injury (10 mm). A 12 mm x 7 mm sheet of small intestine submucosa (SIS) was cut and sutured to form a hollow tube having final dimensions of 12 mm length x 2 mm diameter using three non-continuous knots (9–0 sutures). Surgical methods for implantation are described below.

Animals were anesthetized with isoflurane and body temperature was monitored through the duration of the procedures to minimize suffering. For the crush injury, the sciatic nerve was exposed by creating a longitudinal incision down the midline of the posterior thigh and carefully splitting the gluteal muscles. The sciatic nerve was crushed 4 mm distal to the ligaments of the greater sciatic foramen for 30 s using #5 forceps, resulting in an approximate 0.5 mm wide defect. The injury was created with the minimum force required to fully close the forceps by the surgeon. For the 3-week transection group, a 10 mm section of sciatic nerve was removed (4 mm distal to the greater sciatic foramen) and the hollow SIS conduit was sutured to the proximal and distal nerve stumps. The gluteal muscles were reattached, and the skin was closed using 5–0 PGCL resorbable sutures [S-Q518R13]. Animals were sacrificed at their assigned timepoint via pentobarbital over administration; 10 mm long sciatic nerve segments were collected and either (1) minced, placed in Trizol [Invitrogen, 15596018], and stored at -80°C for RNA extraction, (2) minced, aliquoted into PBS, and stored at -20°C for biochemical assessments, or (3) placed directly in 4% paraformaldehyde [Fisher, AAJ19943K2] for immunohistochemical protein analysis.

### 2.2 RNA extraction and polymerase chain reaction (PCR)

Cell lysates were obtained by using a hand sonicator [QSonica, Q55 with 1/8” probe] to homogenize nerve samples in 1 ml Trizol over ice. The homogenized samples were incubated at room temperature for 5 min before adding 200 μL chloroform [Crescent Chem Co Inc, 67-66-3] per mL of Trizol. Samples were shaken for 15 s by hand, left to incubate for 3 min, then centrifuged at 12,000x g at 4°C for 15 min. The aqueous, RNA-containing phase was isolated, then precipitated in 0.75 mL of isopropyl alcohol per 1 mL of Trizol. Samples were mixed and incubated at room temperature for 10 min before centrifuging at 12,000x g for 15 min at 4°C. The supernatant was removed, and the RNA pellet was washed with 1 mL of 75% ethanol.

Samples were centrifuged at 10,000x g for 5 min at 4°C. The supernatant was discarded, and the RNA pellet was dried in air before resuspending in RNase-free water. RQ1 RNase-Free DNase [Promega, M6101] was used to treat RNA samples according to the manufacturer’s instructions. RNA cleanup was performed using the RNeasy® MinElute® Cleanup kit [Qiagen, 74204] and RNA concentration and purity were determined with a NanoDrop spectrophotometer. Sample purity was ensured to have 260/280 ratios between 1.8 and 2.1. RNA was converted into cDNA using the High-capacity cDNA Reverse Transcription kit [ThermoFisher 4368814] according to the manufacturer’s instructions.

PCR primers were designed using NCBI Primer-BLAST [27] for the following genes: HYAL1, HYAL2, CD44, and RICTOR (Table 1). Hyaluronidase species HYAL1 and HYAL2 were chosen as targets, as they have been widely characterized in other somatic tissues [28, 29]. CD44 was chosen as a target as it is the principal receptor for HA binding [30]. RICTOR was chosen as a housekeeping gene because it exhibits insignificant changes in gene expression following peripheral nerve injury [31]. Each primer was optimized for appropriate amplification by determining its melting temperature (T_m_). Quantitative reverse transcription PCR (RT-qPCR) was performed using 30 μL reaction volumes with PowerUp SYBR Green master mix [ThermoFisher, A5742] in a QuantStudio 3 Real-Time PCR system [Applied Biosystems] with VeriFlex temperature zones of 61°C and 60°C for their respective optimized primers. Gene expression was quantified using a comparative cycle threshold method ΔΔCt (n = 6). Each biological replicate’s cycle threshold (Ct value) was determined by averaging across three technical replicates. To obtain ΔCt values for the genes of interest, the Ct value for each biological replicate was normalized to the RICTOR value. Then, this ΔCt value was normalized to the average ΔCt of fresh tissue control samples to determine ΔΔCt values.

**Table 1:** *Primer sequences for target genes*.

Gene	Species	Forward	Reverse	T_m_(C°)
HYAL1	Rat	GCC CAT AAT GCC CTA CGT CCA	TGG CTT GGC ATG ACT CCT TG	60
HYAL2	Rat	GGA GCG GGC TTA GCT GGT A	GGG CTA CAG GAA GTG TCA CC	61
CD44	Rat	TCC ACC CCA ACG CTA TCT GT	TTG GAT GCG AGG AGG ATA TAC A	61
RICTOR	Rat	ATG GCC TTC AGG AGC GAG AC	CGA AGG GCT ACC ACC TCT GG	61

### 2.3 Gene arrays

Rat Extracellular Matrix and Adhesion Molecules RT2 Profiler PCR Arrays were purchased and used per the manufacturer’s instructions [Qiagen, PARN-013ZA]. cDNA was mixed with RT2 SYBR Green ROX qPCR master mix [Qiagen, 1065855] and RT-qPCR was performed using 25 μL reaction volumes in a QuantStudio 6 Flex system. Gene expression was quantified using ΔΔCt. The geometric mean of the Ct values of the manufacturer provided housekeeping genes was used to provide a baseline Ct value. To obtain ΔCt, the housekeeping baseline value was subtracted from the Ct value obtained from each sample target (n = 1). To obtain ΔΔCt, the ΔCt value of the fresh control target was subtracted from the ΔCt of each target with all genes and experimental groups.

### 2.4 Immunohistochemical analysis

The harvested whole tissue samples were immediately fixed in 4% paraformaldehyde for 24 h and washed with 1X PBS once daily for 3 d, and stored in 30% sucrose [ThermoFisher, S550] and 0.5% sodium azide [Sigma, S2002] in 1X PBS at 4°C for at least 24 h before cryosectioning. All samples were embedded in optimal-cutting-temperature compound [Electron Microscopy Sciences, 6255001] and longitudinally sectioned (10 μm) with a Leica CM1950 cryostat. Sections were deposited onto gelatin-coated glass slides and dried overnight. Slides were stored at -80°C until subsequent staining.

Slides were removed from -80°C and warmed at 37°C for 2 h. Slides were blocked with blocking buffer consisting of 3% goat serum [Sigma, G9023] and 0.3% Triton X-100 [Sigma, 93443] in 1X PBS for 1 h. Slides were incubated with primary antibodies (1:500), rabbit anti-HYAL1 [LS Bio, LS-C409001] and mouse anti-βIII tubulin (pan-neuronal marker) [Abcam, ab7751] or rabbit anti-HYAL2 [Abcam, ab68608] and mouse anti-S100 (Schwann cells) [Abcam, ab14849], overnight at 4°C. Slides were washed with 1X PBS 3x10 min at room temperature, followed by secondary antibody incubation with (1:250) Cy5 anti-mouse [Jackson ImmunoResearch, 115-175-146] and (1:250) Cy3 anti-rabbit [Jackson ImmunoResearch, 611-65-215] for 2 h. Slides were washed with 1X PBS 3x10 min, labelled with DAPI (1:1000 in water) for 5 min, and washed once with 1X PBS for 10 min. Lastly, slides were coverslipped with Fluoromount-G [SourthernBioTech, 0100–01] and then sealed with clear polish around the edges of the coverslip. Fluorescence tiled imaging of the whole nerve section was conducted on a Zeiss Axioimager Z2. Staining and imaging procedures were repeated for all samples to acquire three technical replicates. Average fluorescence intensity for each section was measured within a region of interest at the proximal end, where the injury was administered.

### 2.5 Hyaluronan concentration measurement

Hyaluronan content was determined using the Purple-Jelley Hyaluronan Assay, per the manufacturer’s recommendations [Biocolor, H1000]. Briefly, tissues were removed from -20°C, weighed and homogenized in 50 mM Tris-HCl (pH 7.5) and 5% v/v proteinase K with a hand sonicator. Proteins were removed by incubating samples overnight at 55°C, centrifuging extracts at 13,000x g for 10 min and transferring the supernatant to a new microcentrifuge tube. Glycosaminoglycan (GAG) precipitation reagent was added to the supernatants, mixed, and left undisturbed for 15 min before centrifuging at 13,000x g for 10 min and discarding the supernatant. Sample residues were resuspended in 360 μL water through vortexing and pipetting. Concentrated sodium chloride (40 μL) and cetylpyridinium chloride (95 μL) were added to the samples, left undisturbed for 30 min at room temperature, then centrifuged at 13,000x g for 10 min. Supernatants were transferred to a new microcentrifuge tube and the protocol was repeated, starting from the addition of GAG precipitation reagent to the retained supernatants.

After repeating the protocol and transferring the resulting supernatant to a new microcentrifuge tube, GAG precipitation reagent was added to the supernatant, mixed, and left undisturbed for 15 min. Samples were centrifuged at 13,000x g for 10 min and the residues were retained. Ethanol was added directly to the residues without mixing and centrifuged again at 13,000x g for 5 min. The resulting supernatant was discarded, and the tubes were drained dry on an absorbent paper. Water (100 μL) was used to resuspend the resulting hyaluronan pellet and rehydrated for at least 30 min before transferring 20 μL of each sample in duplicate to a microplate with 200 μL Purple-Jelley dye reagent. The plate absorbance was read with a spectrophotometer at 655 nm.

The absorbance measurements were converted to concentrations using a standard curve. The amount of HA per mg of wet tissue was determined by dividing the concentration of hyaluronan by the initial wet weight of tissue digested.

### 2.6 Hyaluronidase activity measurement

Enzymatic activity was determined using the Hyaluronidase Assay Kit [AMSBIO, Ra003-01-HAK]. Briefly, tissues were removed from -20°C and thawed on ice, weighed, and homogenized at 15 mg/ml in base buffer with a hand sonicator, then centrifuged at 2000x g for 2 min to separate the supernatant for assay measurement. The supplied microwell plate was rehydrated with 1X PBS for 30 min at room temperature and washed with distilled water. Reaction buffer and sample supernatant (50 μL of each) was added to the wells and incubated for 1 h at 37°C on a shaker plate (300 rpm). Samples were added to the wells in duplicate. Following the reaction, the microplate was washed with 0.1% v/v Tween in PBS four times, followed by two washes with distilled water. Manufacturer provided streptavidin-horseradish peroxidase (100 μL) was added to the wells and incubated for 50 min at 37°C on a shaker plate (300 rpm). The microwell plate was washed five times with 1X PBS then dried by tapping the plate on a wet absorbent paper. Room temperature peroxidase substrate (100 μL) was added to each well and the absorbance was read at 650 nm until the optical density (OD) reached 0.25. The reaction was promptly stopped by adding 100 μL 0.12M HCl to each sample well of the microplate. The absorbance was read at 450 nm within 5 min of adding the stop solution. The enzymatic activity was calculated by determining R from the experimental OD and OD_max_ determined from negative control wells:

$$R=\left(\frac{OD}{{OD}_{max}}\right)*500$$ Eq 1

R was then substituted to find the rate of HA removed:

$$ng\;HA\;removed\;per\;min=\frac{500-R}{60}$$ Eq 2

Enzymatic activity (Units) was determined by converting the rate of “ng HA removed per min” to “μmol HA removed per min,” where volume of the reaction was 50 μL and the molecular weight of one HA monomer is 380 g/mol:

$$Units=\mu mol\;HA\;removed\;per\;min=\frac{ng\;HA\;removed\;per\;min}{\left(0.05*380\right)}$$ Eq 3

Lastly, specific activity, defined as the units per milligram of protein (U/mg), was determined by dividing the activity values determined from Eq 3 by the concentrations of HA determined from 2.5.

### 2.7 Statistical analyses

All statistical analyses were performed using JMP Pro 14 and R-Studio to determine differences between nerve crush groups and 3-week injury models. Shapiro-Wilk and Kolmogorov-Smirnov tests were used to test the normality of the experimental group distributions of gene expression results [32]. Because of the non-normality of the data, Kruskal-Wallis tests were used to determine statistically significant differences between the experimental group medians [33]. Pairwise comparisons were made post-hoc using the Conover-Iman Multiple Comparisons Test [34], with a single-step p-value adjustment. Differences were deemed statistically significant at a significance level α = 0.05. Statistical differences between experimental groups were determined with 1-way ANOVA for immunohistochemical analysis, hyaluronan measurements, and hyaluronidase activity measurements. Analyses were followed by Tukey’s Honestly Significant Difference posthoc test [35] for multiple comparisons, with a 95% family-wise confidence level.

Fold change data are graphed in box-and-whisker plots as minimum observation, lower 25% quartile (Q1), median, upper 75% quartile (Q3), and maximum observation. Outliers were determined as values greater than Q3 plus 1.5 times the interquartile range (IQR), or values less than Q1 minus 1.5 times the interquartile range. All other data are represented as the mean ± standard deviation (SD).

## 3. Results

### 3.1 Gene and protein expression of hyaluronidases

**Fig 1:**
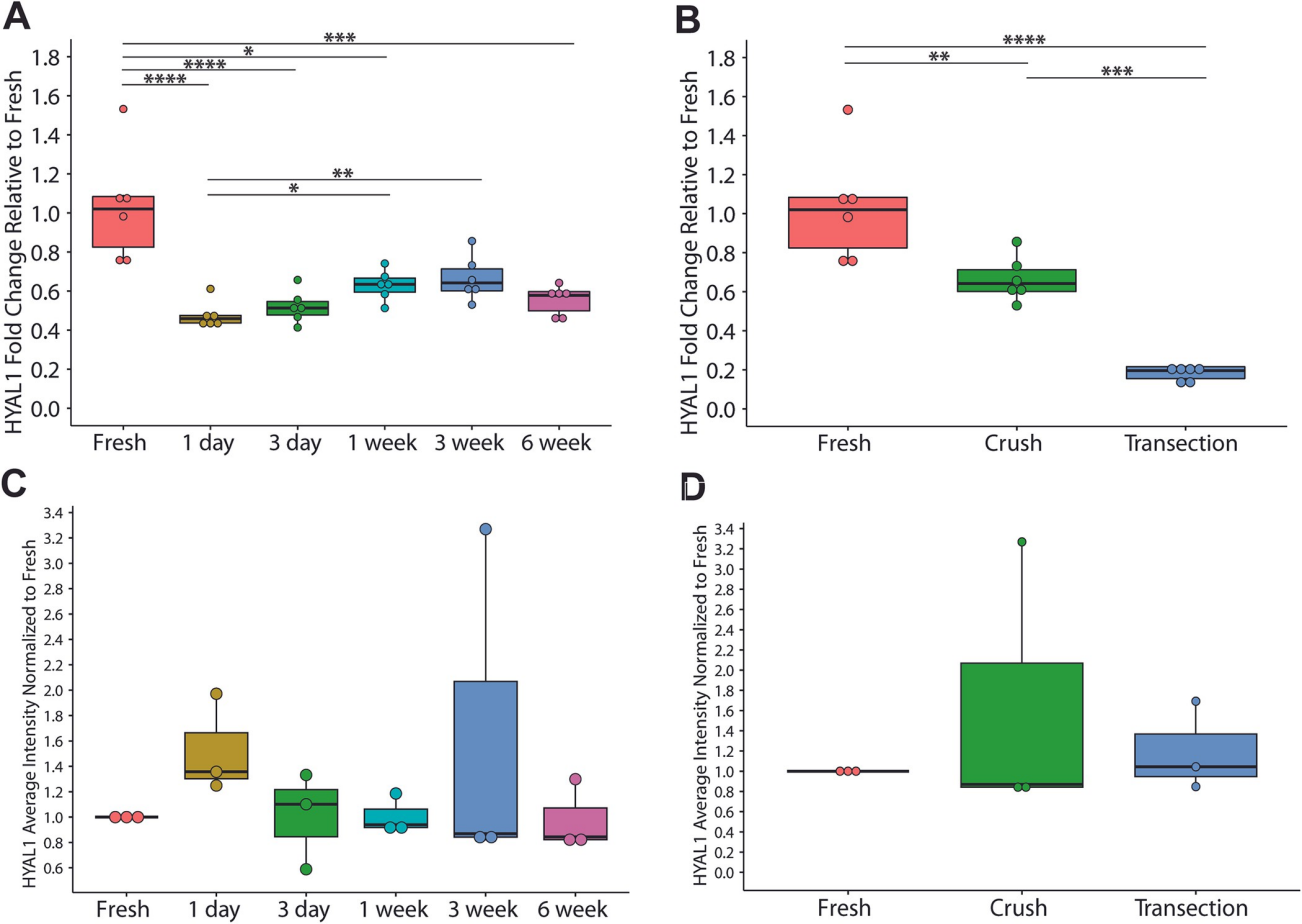
Gene expression of HYAL1 comparing A) timepoints after nerve crush injury and B) comparing nerve injury models at 3 weeks after injury (n = 6). Gene expression data show an overall downregulation of HYAL1 after injury with the greatest expression at 3 weeks after crush injury. Significant differences are observed between injury models, with transection exhibiting the greatest downregulation at 3 weeks. Protein expression of HYAL1 quantified through immunohistochemical analysis across C) timepoints after nerve crush injury and D) nerve injury models 3 weeks after injury (n = 3). There appear to be no differences in protein expression between timepoints or injury models. * p<0.05, ** p<0.01, *** p<0.001, **** p<0.0001.

All crush timepoints in Fig 1A showed a significant overall downregulation of HYAL1 compared to fresh, with the exception of 3 weeks post-injury, where HYAL1 was detected at levels similar to uninjured tissue (fresh vs 1 day: p = 0.0000025, fresh vs 3 day: p = 0.00005, fresh vs 1 week: p = 0.04690, fresh vs 3 week: p = 0.11041, fresh vs 6 week: p = 0.00031). Between 1 day and 3 weeks (1 day vs 1 week: p = 0.01060, 1 day vs 3 week: p = 0.00380), there was a gradual and significant increase in HYAL1 gene expression to levels equivalent to that in fresh nerve (fresh vs. 3 week: p = 0.11041), followed by a return at 6 weeks to expression observed at 1 day (1 day vs 6 week: p = 0.50884). HYAL1 protein expression did not appear to change across crush time points compared to fresh tissue, however, there was a slight, but non-significant, increase in HYAL1 immunostaining at 3 weeks (Fig 1C).

Comparing across groups in Fig 1B (3 weeks after injury), significant differences were observed, where transection exhibited a downregulation of HYAL1 compared to crush and fresh tissue (fresh vs crush: p = 0.00243, fresh vs. transection: p = 0.00000054, crush vs. transection: p = 0.00053). There were no observable differences in protein expression between injury models at 3 weeks (Fig 1D).

**Fig 2:**
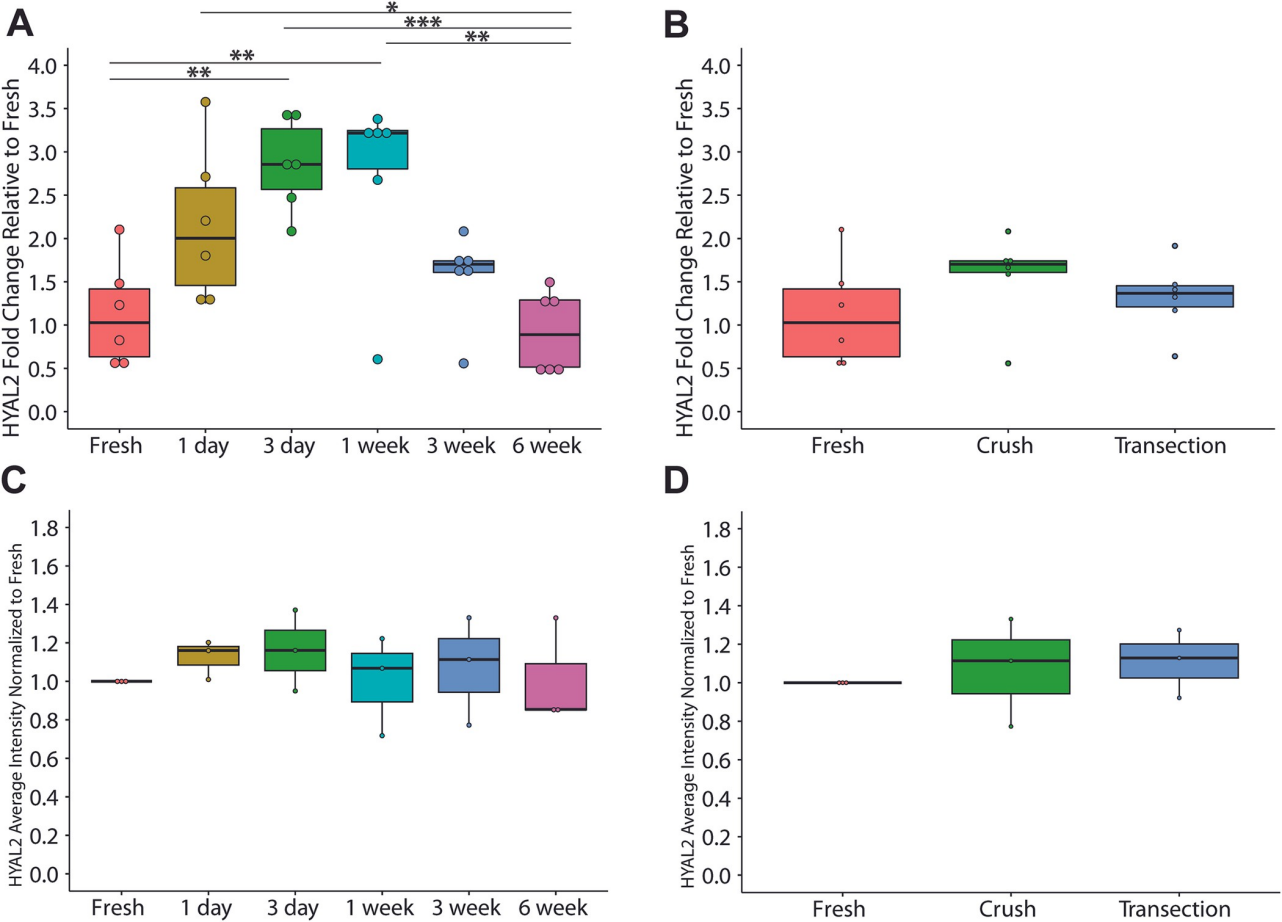
Gene expression of HYAL2 comparing A) timepoints after nerve crush injury and B) comparing nerve injury models 3 weeks after injury (n = 6). Temporal changes are observed following nerve crush with the greatest expression between 3 days and 1 week. No significant differences in gene expression are observed between injury models at 3 weeks. Protein expression of HYAL2 quantified through immunohistochemical analysis comparing C) timepoints after nerve crush injury and D) comparing nerve injury models 3 weeks after injury (n = 3). No significant differences observed between crush timepoints or injury models. * p<0.05, ** p<0.01, *** p<0.001.

Gene expression data of HYAL2 after a nerve crush (Fig 2A) showed an increase in fold change following injury with the greatest changes from fresh observed between 3 days and 1 week (fresh vs 3 day: p = 0.00219, fresh vs 1 week: p = 0.00930), measured fold change returning to levels observed in fresh nerve by 3 weeks (fresh vs 3 week: p = 0.80463). There were no observable differences in HYAL2 gene expression between fresh nerve tissue and either crush or transection injury at 3 weeks (Fig 2B). There were no observable differences detected in HYAL2 protein data between crush timepoints (Fig 2C) or injury models at 3 weeks (Fig 2D).

### 3.2 HA quantification and CD44 gene expression

**Fig 3:**
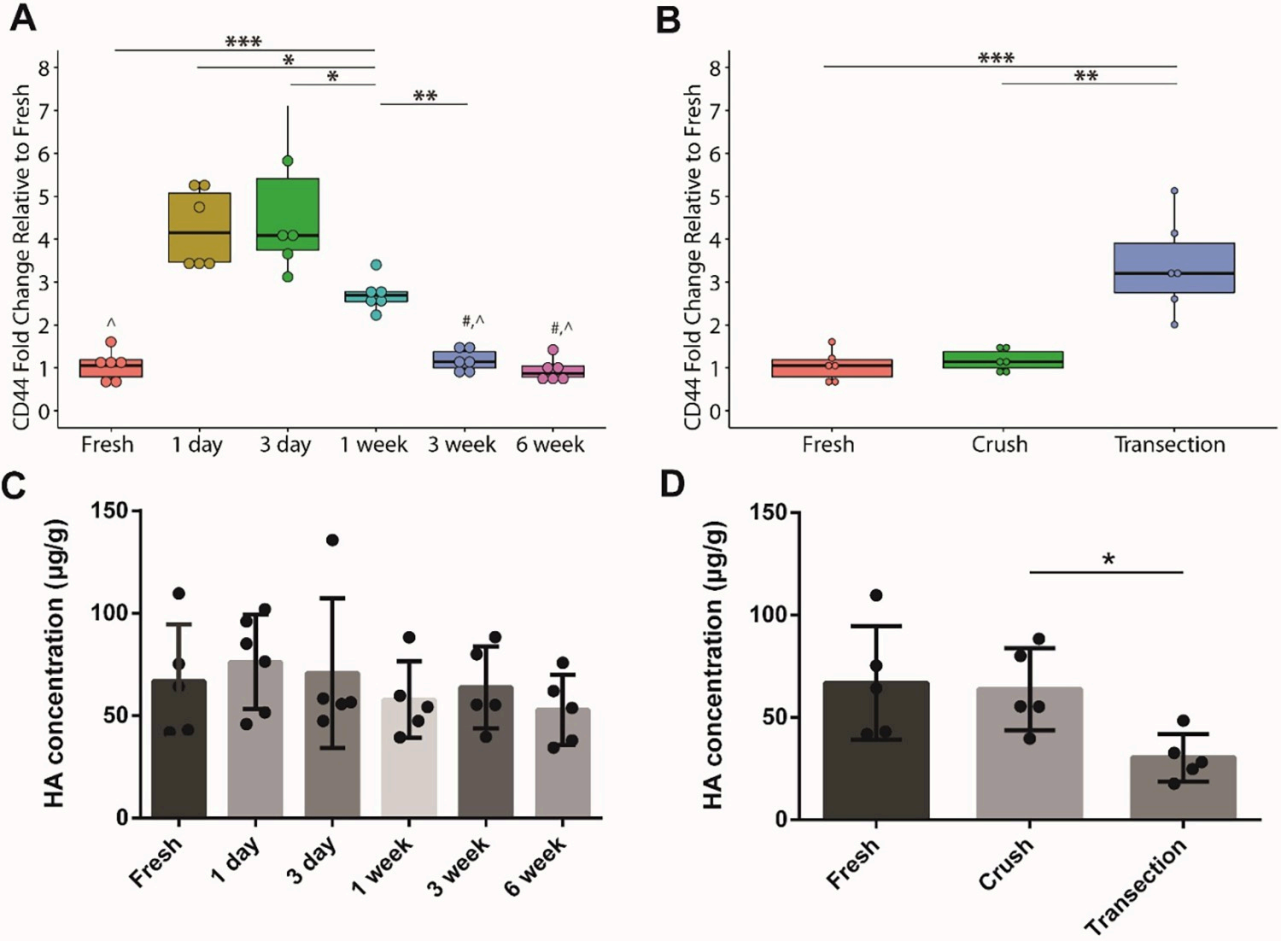
Gene expression of CD44 comparing A) timepoints after nerve crush injury and B) comparing nerve injury models at 3 weeks after injury (n = 6). There appear to be temporal changes after nerve crush with the greatest CD44 gene expression observed at 3 days after crush. Between injury models at 3 weeks, CD44 is highly expressed following transection at 3 weeks compared to other groups. HA quantification comparing C) timepoints after nerve crush injury and D) comparing nerve injury models 3 weeks after injury (n = 6). No differences observed in HA concentration between crush timepoints, while there is a significantly lower HA concentration measured after transection at 3 weeks. # = not significant to fresh tissue, ^ p<0.0001 to 1 day and 3 day, * p<0.05, ** p<0.01, *** p<0.001, **** p<0.0001.

Gene expression analyses of CD44 following nerve crush (Fig 3A) demonstrated a significant fold change from fresh tissue 1 day after injury with levels remaining elevated through 1 week after injury (fresh vs 1 day: p = 0.00000011, fresh vs 3 day: p = 0.0000004, fresh vs 1 week: p = 0.00045). Expression eventually returned to healthy conditions after 3 weeks (fresh vs 3 week: p = 0.88241). Comparatively, after nerve transection (Fig 3B), gene expression levels remained significantly elevated compared to crush or fresh nerve samples (fresh vs crush: p = 0.7568, fresh vs transection: p = 0.0003, crush vs transection: p = 0.0012).

After nerve crush injury, there appeared to be an increase in HA concentration, however, differences are not significant (Fig 3C). Comparing across nerve injury models at 3 weeks, there was a significant decrease in HA concentration after nerve transection compared to fresh tissue or crush injury samples (Fig 3D).

### 3.3 Gene analysis of ECM components in peripheral nerve

**Fig 4:**
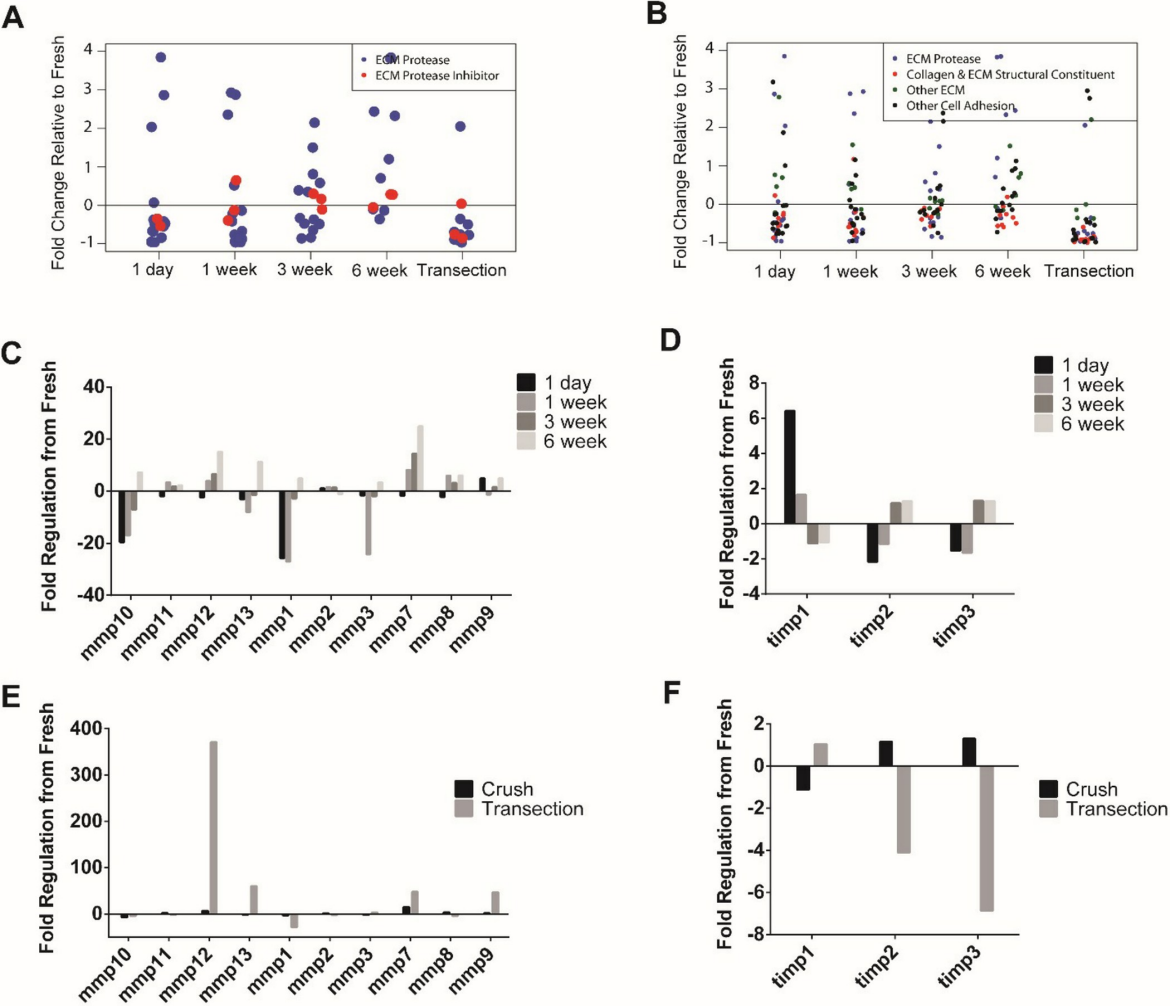
Gene array analysis of various ECM proteins, proteases, and adhesion molecules after nerve injury (n = 1, no statistical power). Overlay of ECM proteases with A) protease inhibitors and B) ECM molecules including collagens, structural constituents, and cell adhesion molecules. Temporal patterns of C) matrix metalloproteases (mmps) and D) tissue inhibitors of metalloproteinases (timps) after nerve crush show differences in patterning across different molecular species. Comparison of fold regulation compared to Fresh tissue of E) mmps and F) timps 3 weeks after crush versus transection show differences between injury models.

Gene arrays were used to analyze the presence of various ECM components within nerve tissue after injury. Fold changes of ECM proteases, protease inhibitors (Fig 4A), collagens, cell adhesion molecules, and other ECM molecules (Fig 4B) showed a spread of behavior with no distinct patterns of up or downregulation of specific molecule types after injury compared to fresh tissue samples. Patterns of individual matrix metalloproteinases (MMPs) (Fig 4C) and tissue inhibitors of metalloproteinases (TIMPs) (Figs 3 and 4D) after nerve crush injury showed general patterns of either early overexpression followed by downregulation at later time points (after 3 weeks) or vice versa. These patterns of relative gene expression varied based on protein target. Fold regulation of proteases, (Fig 4E) and protease inhibitors (Fig 4F) after 3 weeks in different injury models showed differences in expression between targets where there are either minimal differences (MMP2), inverse differences between injury models (TIMP3), or dramatic changes in either under or overexpression (MMP12).

### 3.4 Hyaluronidase measurements

**Fig 5:**
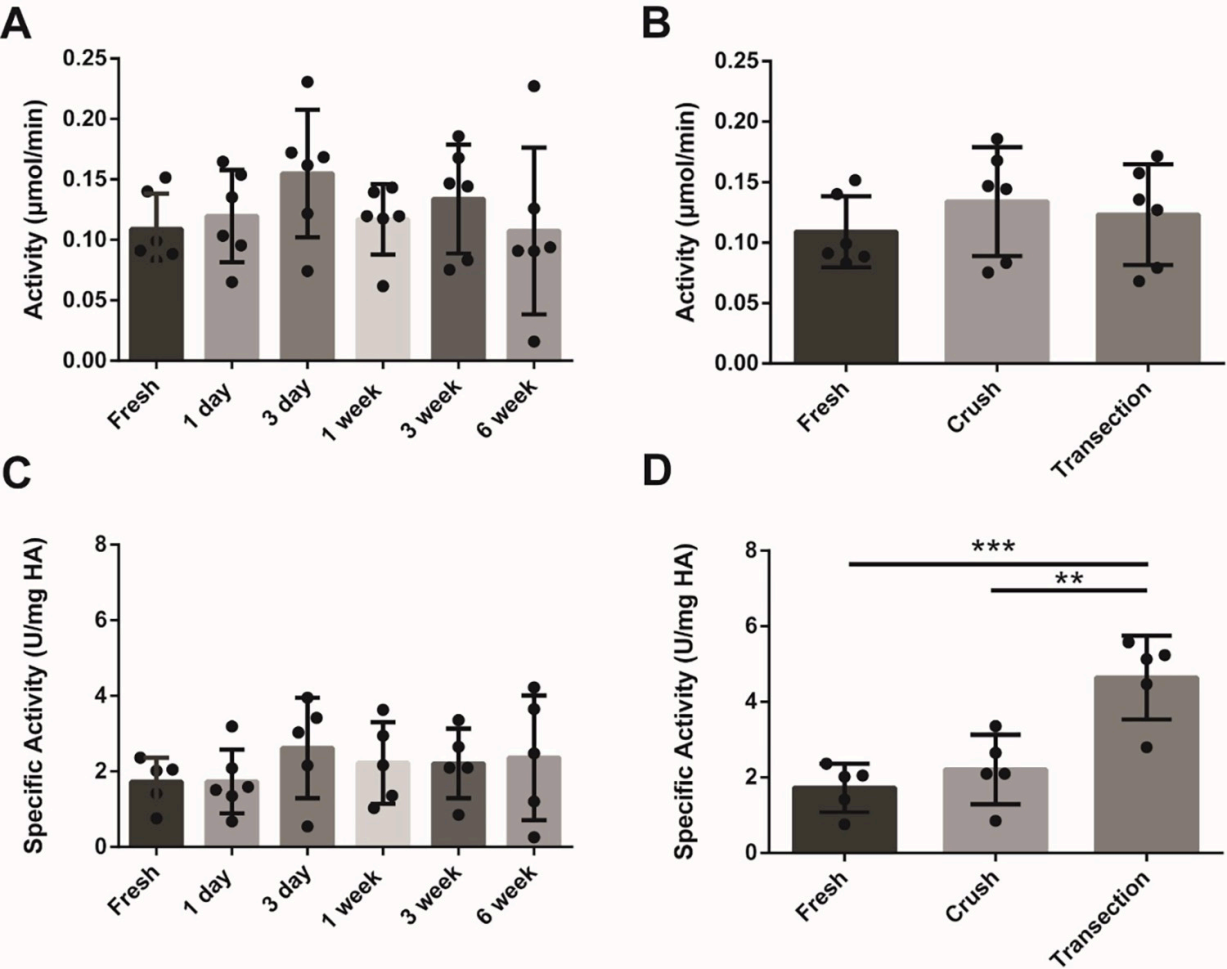
Comparison of hyaluronidase activity following A) nerve crush injury at various timepoints and B) 3 weeks after nerve crush vs. transection injury (n = 6). No significant differences observed between crush timepoints or injury models. Hyaluronidase specific activity measurements comparing A) nerve crush injury at various timepoints and B) 3 weeks after crush and transection injuries (n = 6). No differences in specific activity observed between crush timepoints, although there appears to be a greater specific activity after transection, related to a higher matrix turnover. ** p< 0.01, *** p<0.001.

Measured hyaluronidase activities showed no differences between crush timepoints (Fig 5A) or injury models (Fig 5B) compared to fresh nerve (0.11 μmol/min ± 0.03). Small trends of peak hyaluronidase activity were measured at 3 days (0.15 μmol/min ± 0.06) with a modest peak also observed at 3 weeks (0.13 μmol/min ± 0.04) following crush injury. Specific activity (Fig 5C) of fresh tissue was determined to be approximately 1.72 ± 0.64 U/mg, with no significant differences observed across crush groups. At 3 weeks (Fig 5D), the transection sample had a measured specific activity of 4.64 ± 1.11 U/mg, which was significantly different compared to fresh nerve and crush samples (1.92 ± 0.92 U/mg).

## 4. Discussion

Hyaluronidases have been scarcely quantified in central nervous tissue and have yet to be evaluated in peripheral nerve tissue; thus, in this work we sought to characterize the presence of HA and hyaluronidase species, HYAL1 and HYAL2, temporally after nerve crush injury. Additionally, this work compares differences in expression between crush and transection injury models at 3 weeks after injury to explore differences in injury and regeneration progression.

Previous studies have shown that accumulation of extracellular matrix components after peripheral nerve injury (e.g., chondroitin sulfate proteoglycans (CSPGs) and MMPs) are inhibitory to regeneration [36, 37]. Nerve repair has been enhanced by reducing the accumulation of CSPGs through the administration of catabolic enzymes (i.e., chondroitinase ABC, MMP-2, and MMP-9) [38]. These trends are similar to that observed in the central nervous system, where accumulation of CSPGs and HA contribute to an inhibitory glial scar. Thus, understanding HA and HYAL expression in the peripheral nervous system may be integral to studying disease progression and formulating subsequent therapeutics.

HYAL2 is the main mediator of extracellular high molecular weight HA breakdown, whereas HYAL1 is responsible for intracellular breakdown of HA oligomers smaller than 20 kDa [39]. These roles define their temporal action, where HYAL2 is expressed as a precursor to HYAL1. In these studies, early temporal changes in HYAL2 gene expression after nerve crush injury showed the greatest changes with respect to fresh between 3 days to 1 week after injury. After nerve crush and transection injuries, extracellular changes are expected to dissipate by 3 weeks once cells have effectively restabilized the injury space [40]. Accordingly, there were no differences in HYAL2 gene expression between fresh tissue controls, crush, or transected injury models at 3 weeks. As expected from their respective roles, HYAL1 showed the greatest changes in expression between 1 week and 3 weeks, compared to 1 day. These results demonstrate the temporal roles of the two dominant hyaluronidase species found in somatic tissues following injury.

Although HYAL1 shows a gradual increase in gene expression following 1 day after nerve crush injury, HYAL1 appears to be downregulated overall from fresh tissue at all time points following nerve crush, even out to 6 weeks. Moreover, there was an even greater downregulation of HYAL1 following transection compared to crush injury at 3 weeks. These behaviors are unexpected because HYAL1 levels never returned to that of native tissue, even after complete nerve repair had occurred at 6 weeks following crush. HYAL3 is encoded on the same chromosome as HYAL1 and 2 and is thought to contribute to housekeeping HA catabolism, though it has been far less characterized in tissue [8]. Studies that have sought to evaluate the role of HYAL3 have generally found that it has negligible enzymatic activity, however it may instead have linked roles to HYAL1 expression. There are similarities between the two species as both are acid-active hyaluronidases and both are found to be expressed within cytoplasmic vesicles, suggesting an intracellular mechanism of degradation [41]. It has also been suggested that HYAL3 may participate in augmenting or compensating for a deficiency in HYAL1. For example, several studies evaluating the expression of HYALs 1, 2, and 3 in various human tissues found that HYAL3 was expressed in brain, whereas HYAL1 and 2 saw little to no expression in brain tissue [28, 42]. For the purposes of this study, the two most abundant HYALs were evaluated, however these differential observations in nervous tissue motivate the need to characterize other HYAL species, such as HYAL3, in peripheral nerve tissue to determine if a decrease in HYAL1 expression could be linked to compensation by other enzymes.

Gene expression analyses showed an overexpression of CD44, the principal receptor for HA binding, immediately following nerve crush with levels remaining elevated through 1 week after injury. Expression eventually returned to healthy conditions after 3 weeks. HA concentration quantification corroborated trends seen in nerve crush CD44 gene analysis, where the greatest differences from fresh tissue were observed immediately after injury and dissipated over time. Similar to trends observed in extracellular HYAL2 patterning, it is expected that by 3 weeks, extracellular matrix changes following a crush injury would return to a quiescent state once the tissue has been restabilized in the earlier wound healing steps. Interestingly, after nerve transection, CD44 remained significantly elevated 3 weeks after injury. Complete transection of epineurial and endoneurial structures requires repopulation of host cells and reestablishment of tissue matrix, resulting in a delayed wound healing cascade compared to crush injury [43]. Thus, the overexpression of CD44 at 3 weeks following transection compared to a crush injury is likely attributed to this reestablishment of native matrix components, including HA. Overexpression of CD44 in the 3-week transection group was also correlated to a decrease in HA concentration. This could be attributed to incomplete regeneration, where HA has not yet been fully deposited because the wound healing cascade is still occurring. Additionally, although HA binds to CD44, CD44 itself also binds to other proteoglycans such as heparin sulfate and chondroitin sulfate [44, 45]. This may explain the discrepancy between gene expression analysis of CD44 and measured HA concentration, where there may be CD44 activation by other molecules instead of solely by HA [44, 45]. These data motivate the need to perform a more detailed study examining HYAL and HA temporal patterning in a transection model. Peripheral nerves have the capability to spontaneously regenerate at sub-critical gap lengths, thus the information provided by a study investigating differences in HYALs and HA could be valuable to further elucidating molecular roles in regeneration between injury models (i.e., crush, subcritical transection, critical transection), especially considering the implication of other inhibitory proteoglycans such as chondroitin sulfate that also bind to CD44 [36, 46].

Gene arrays were performed to gain a better understanding of the ECM landscape following peripheral nerve injury. Although there was not enough statistical power to determine significance of a specific protein across experimental groups, it can generally be concluded that different species of proteins and enzymes, even within the same general class, have different patterns of expression (S1 Table). This supports the difference in trends observed between HYAL1 and HYAL2 gene behavior and further motivates the need to investigate other ECM molecules during peripheral nerve regeneration.

Although there were no observable differences in the protein expression of either tested hyaluronidase species, compared to fresh tissue and regardless of injury model, protein expression intensity measurements for HYAL2 and HYAL1 had modest upticks at 3 days and 3 weeks after nerve crush, respectively. These protein trends corroborate with those observed in gene expression where the greatest changes were seen at 3 days and 3 weeks after nerve crush in HYAL2 and HYAL1, respectively. Differences in protein expression from gene expression may be attributed to post-transcriptional modifications, however it is important to note that semi-quantitative immunohistochemical protein analysis was performed in triplicate on only one biological sample to conserve animal numbers (S1 and S2 Figs). Thus, future work should ideally include a deeper exploration into protein expression, specifically, utilizing more quantitative methods such as Western blot analyses.

Although measured hyaluronidase activities showed no significant differences across crush timepoints compared to fresh nerve (0.11 μmol/min), there appeared to be modest peaks in activity measured at 3 days and 3 weeks, aligning with the combined protein and gene expression data for both HYAL species. Specific activity measurements also showed no differences between crush groups, although there appeared to be differences between injury models at 3 weeks, aligning with observed differences in HA concentration measurements. A smaller HA concentration and increased specific activity in the transection group compared to crush and fresh at 3 weeks provided further evidence of wound healing differences between injury models.

While hyaluronidase activity has generally been difficult to measure because of its low endogenous presence (between 1 and 6.3 μg/mg protein in liver and kidney, respectively), specific activity in fresh nerve tissue measured from this study fall within the range of values previously reported for more metabolic tissues [47]. In general, there were no observable differences in enzymatic activity between crush timepoints; however, comparisons between crush and transection injury models at 3 weeks showed emerging differences, thus motivating the recommendation for additional studies investigating temporal changes in a transection model. Finally, this work provides a quantitative measure of hyaluronidase activity in peripheral nerve that can be used for the development of degradable HA-based scaffolds.

## 5. Conclusion

This work provides empirical information important to understanding the enzymatic environment of peripheral nerve tissue following injury. This information is particularly valuable for tissue-engineered applications because it provides quantitative data that can be used to model the behavior of the peripheral nerve environment, as well as the breakdown of hyaluronan-based therapeutics.

Prior to this study, there were no bodies of work characterizing hyaluronidase expression or activity in peripheral nerve tissue; thus, this research focused primarily on changes in protease gene expression following nerve crush injury. A meaningful continuation of this work should include more quantitative protein analyses after nerve crush, such as Western blot analyses or a more in depth immunohistochemical study. Another limitation of the work presented is that it does not provide a full picture of hyaluronidase activity following a transection injury where hyaluronan-based therapeutics would be most likely to be administered. Future studies will focus on characterizing temporal differences of hyaluronidase expression following nerve transection injury. Other future directions could include investigating other proteoglycans and enzymes found in nerve in comparison to hyaluronan. A future study could also specifically investigate the roles of exogenous hyaluronidase on nerve repair as it has been shown that administration of certain enzymes can improve regeneration [48, 49]. Characterizing the temporal patterning of ECM components could elucidate the limitations of neural regeneration at critical gap length.

## Supporting information

S1 Fig HYAL1 immunohistochemical micrographs. (TIF)

S2 Fig HYAL2 immunohistochemical micrographs. (TIF)

S1 Table Complete ECM gene array table. (XLSX)
