# Supplementary figures and images for: Temporal characterization of hyaluronidases after peripheral nerve injury
Source: PLoS One. 2023 Aug 24;18(8):e0289956. doi: 10.1371/journal.pone.0289956 (PMC10449126; doi:10.1371/journal.pone.0289956)

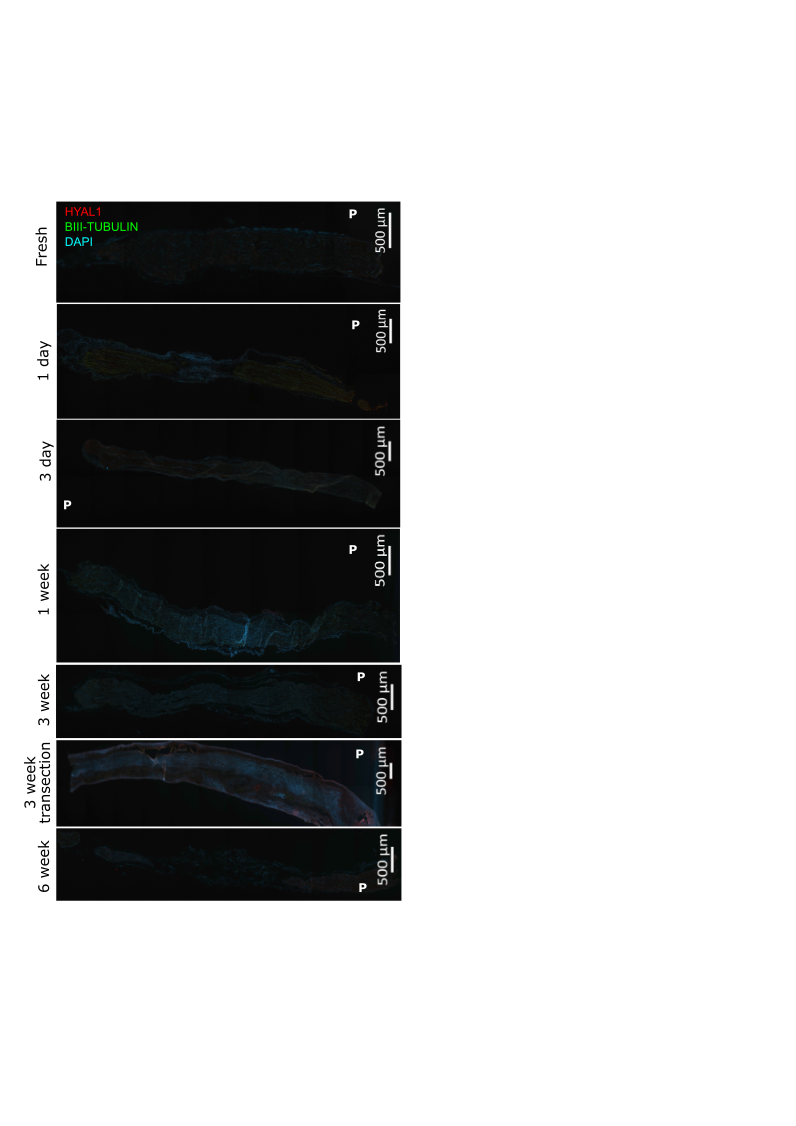

Supplement: S1 Fig — (TIF) [file pone.0289956.s001.tif]

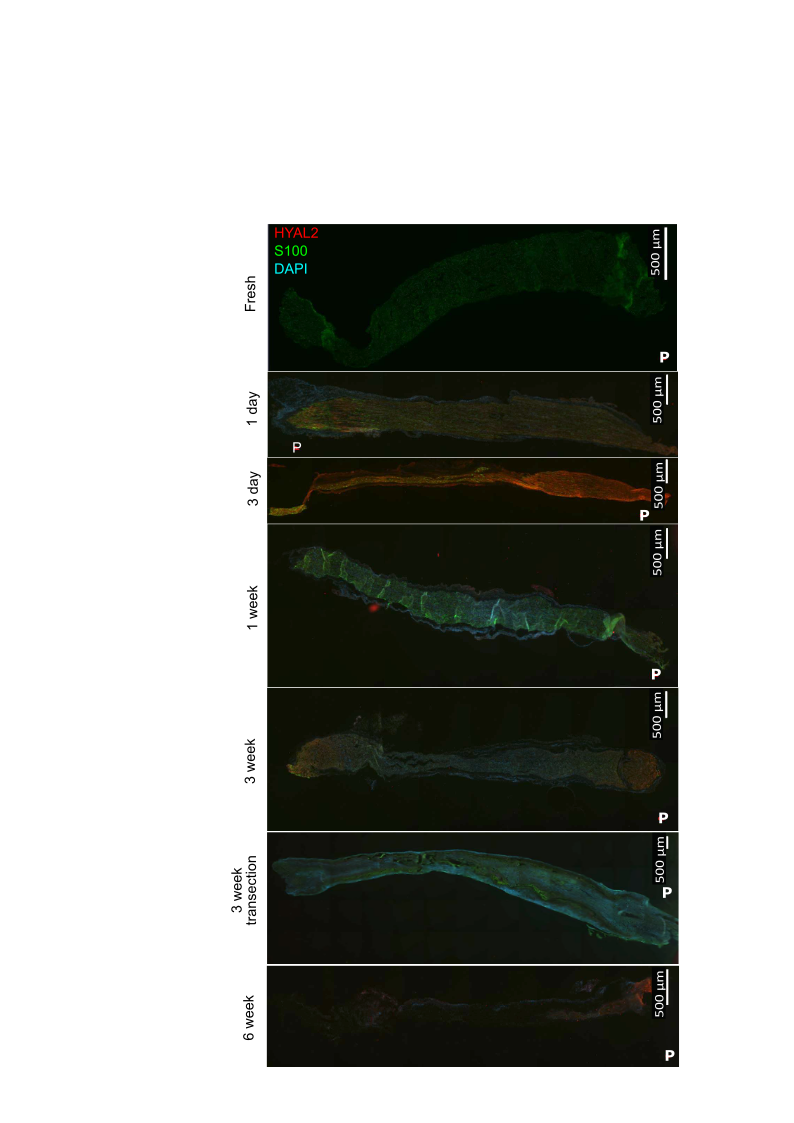

Supplement: S2 Fig — (TIF) [file pone.0289956.s002.tif]
